# COVID-19: Insights into Potential Vaccines

**DOI:** 10.3390/microorganisms9030605

**Published:** 2021-03-15

**Authors:** Ke-Yan Loo, Vengadesh Letchumanan, Hooi-Leng Ser, Siew Li Teoh, Jodi Woan-Fei Law, Loh Teng-Hern Tan, Nurul-Syakima Ab Mutalib, Kok-Gan Chan, Learn-Han Lee

**Affiliations:** 1 Novel Bacteria and Drug Discovery Research Group (NBDD), Microbiome and Bioresource Research Strength (MBRS), Jeffrey Cheah School of Medicine and Health Sciences, Monash University Malaysia, Bandar Sunway 47500, Malaysia; ke.loo@monash.edu (K.-Y.L.); vengadesh.letchumanan1@monash.edu (V.L.); ser.hooileng@monash.edu (H.-L.S.); jodi.law1@monash.edu (J.W.-F.L.); loh.teng.hern@monash.edu (L.T.-H.T.); 2 School of Pharmacy, Monash University Malaysia, Bandar Sunway 47500, Malaysia; teoh.siew.li@monash.edu; 3 Clinical School Johor Bahru, Jeffrey Cheah School of Medicine and Health Sciences, Monash University Malaysia, Johor Bahru 80100, Malaysia; 4 UKM Medical Molecular Biology Institute (UMBI), UKM Medical Centre, Universiti Kebangsaan Malaysia, Kuala Lumpur 56000, Malaysia; 5 Division of Genetics and Molecular Biology, Institute of Biological Sciences, Faculty of Science, University of Malaya, Kuala Lumpur 50603, Malaysia; 6 International Genome Centre, Jiangsu University, Zhenjiang 212013, China

**Keywords:** COVID-19, coronavirus (CoV), vaccines, clinical trials, passive immunotherapies

## Abstract

People around the world ushered in the new year 2021 with a fear of COVID-19, as family members have lost their loved ones to the disease. Millions of people have been infected, and the livelihood of many has been jeopardized due to the pandemic. Pharmaceutical companies are racing against time to develop an effective vaccine to protect against COVID-19. Researchers have developed various types of candidate vaccines with the release of the genetic sequence of the SARS-CoV-2 virus in January. These include inactivated viral vaccines, protein subunit vaccines, mRNA vaccines, and recombinant viral vector vaccines. To date, several vaccines have been authorized for emergency use and they have been administered in countries across the globe. Meanwhile, there are also vaccine candidates in Phase III clinical trials awaiting results and approval from authorities. These candidates have shown positive results in the previous stages of the trials, whereby they could induce an immune response with minimal side effects in the participants. This review aims to discuss the different vaccine platforms and the clinical trials of the candidate vaccines.

## 1. Introduction

In December 2019, the first cases of coronavirus disease 2019 (COVID-19) were reported as pneumonia with an unknown etiology in Wuhan, China [1]. Approximately ten days after the reports, the outbreak was determined to be caused by the novel virus known today as severe acute respiratory syndrome coronavirus 2 (SARS-CoV-2) [2]. A global pandemic was later declared in March by the World Health Organization (WHO) following the disease’s rapid spread across nations in a short period [2]. As this article is being written, the virus has spread throughout 235 countries, infecting more than 112 million people worldwide and has killed over 2.4 million people according to John Hopkins University’s COVID-19 database [3,4]. Remdesivir was approved by the US Food and Drug Administration (FDA) to treat COVID-19 in patients who are ≥12 years old and weigh at least 40 kg. This antiviral drug is able to prevent the viral genome of SARS-CoV-2 from replicating [5,6]. The virus, SARS-CoV-2, belongs to a group of coronaviruses (CoVs) of the Coronaviridae family, namely, the β-CoVs, which infect mammals [7]. Coronaviruses are pleomorphic enveloped particles with a single-stranded RNA genome. There are fringe projections known as spike (S) proteins on the virus’s surface, which are the main characteristic of these viruses [8,9]. The virus uses the S protein to enter the host cell by interacting with the host cell receptor angiotensin-converting enzyme 2 (ACE2). The viral RNA is released in the host cells and triggers a cascade of events that replicate viral RNA and synthesize structural and non-structural proteins. These proteins are vital in the survival and propagation of the virus. Structural proteins include the spike (S) glycoprotein, small envelope (E) glycoprotein, membrane (M) glycoprotein, nucleocapsid (N) protein, and other accessory proteins; whereas non-structural proteins are categorized as nsp 1 to nsp 16. [10] Consequently, more viral particles are released and reside in the host [7,8].

SARS-CoV-2 is mainly transmitted from human to human via respiratory droplets. When an infected person coughs, sneezes, or talks, another person could be infected if the droplets are inhaled or come into contact with their mucous membranes [11]. The virus primarily targets the respiratory system and causes flu-like symptoms upon exposure, although some patients may appear asymptomatic. In the early stages, the disease presents itself as a mild respiratory infection. Still, as the disease progresses in severity, it may lead to acute respiratory failure, severe complications such as multiorgan failure, and ultimately death [9,12]. It has also been found that there is a greater risk of infection in the elderly, patients with chronic diseases, and immunocompromised patients [9,13]. SARS-CoV-2 shares a similar sequencing identity with the infamous SARS coronavirus (SARS-CoV) and Middle East respiratory syndrome coronavirus (MERS-CoV), but SARS-CoV-2 is relatively more infectious in comparison to other CoVs [7]. First identified in 2003, SARS-CoV was thought to originate from an animal (bat) reservoir, which then spread to humans, causing severe acute respiratory syndrome (SARS) in Guangdong, China. The virus quickly spread globally to 30 countries, infecting 8098 people, and killing 774 [14]. The outbreak was declared over in late July after no new cases were reported [15]. Fast forward to 2012, to the emergence of MERS-CoV, a novel coronavirus, which infected more than 2500 people across 27 countries and caused 881 deaths [16]. Interestingly enough, SARS-CoV-2, SARS-CoV, and MERS-CoV are all suspected of being from animal reservoirs and then transmitted to humans [15,16]. They are all easily transmitted from person to person with close contact, and to date, no vaccines have been licensed to treat them. Even though it has been over 15 years since the first SARS-CoV outbreak, only vaccines based on the inactivated virus, DNA, and soluble proteins based on the S protein have reached clinical stages [17,18]. As for MERS, a DNA vaccine (GLS-5300), measles-vectored vaccine, modified vaccinia virus Ankara-vectored vaccine, and chimpanzee adenovirus-vectored vaccines have entered Phase I clinical trials [19]. This shows that a long time is required in research and development to manufacture a safe and effective vaccine to prevent diseases and outbreaks.

Throughout humankind’s history, vaccinations have played a significant role in eradicating diseases and improving public health since the discovery of the smallpox vaccine by Edward Jenner in 1796 [20]. Ever since then, vaccines have been effective at reducing the incidence rate of infections. There has been a rapid development of a COVID-19 vaccine during the pandemic to protect the health of millions worldwide. A variety of strategies have been implemented in many countries to prevent the virus’s spread [6,21,22,23]. Wearing a face mask when going out and practicing good hygiene by hand washing using soap and water or hand sanitizer have been highly recommended, along with avoiding crowded areas. Other strategies include having health screenings at entry and exit points of airports and train stations to ensure passengers are healthy and free from infection. Moreover, individuals presenting symptoms or are asymptomatic but tested positive are put into quarantine for further treatment to prevent the spread of the virus [6]. However, many may still be susceptible to the respiratory disease without having immunity towards the virus when subsequent waves of infection occur. The rapid increase in morbidity and mortality of the COVID-19 pandemic has led to a drastic shift in the conventional vaccine development paradigm and timelines from a time frame of 10–15 years to 1–2 years [24]. The change in the development time frame could mean researchers cannot ascertain the vaccine-induced protective immunity’s longevity and quality. In an ideal world, a vaccine should have high efficacy, with minimum adverse reactions. It should stimulate both humoral and cell-mediated immune responses while being able to induce life-long immunity in the recipients.

Moreover, the vaccine must be easily administered and remain stable during transport and storage. From an economic standpoint, the process to manufacture the vaccine should be reproducible and inexpensive to be easily translated to developing countries [25,26]. Once an effective vaccine is developed, it is crucial that there is equitable access and distribution of vaccines across all nations to protect the public’s health. Demographics at a higher risk should also be prioritized in receiving the vaccine to prevent further spread and fatalities. Amid this pandemic, researchers are racing against time to find a suitable vaccine for COVID-19 and ensure it is a preventable disease. This review describes vaccine candidates currently in Phase III trials, those with emergency use authorization, and the strategies that were used to develop these vaccines.

## 2. The Vaccine Candidates

Vaccines used in healthcare can be classified into live attenuated vaccines, inactivated vaccines, protein subunit vaccines, toxoid vaccines, conjugate vaccines, recombinant vector vaccines, and nucleic acid-based vaccines [27]. In general terms, vaccines are administered to a host to produce an immune response similar to that produced by a natural infection without resulting in a diseased state. The purpose of introducing a vaccine to the host is to produce immunity and an immunologic memory like that of a natural infection [25,27]. If the host is exposed to a natural condition, later, their immune system will be able to elicit an immune response to fight against the infection. As mentioned earlier, the S protein on the SARS-CoV-2 virus plays an essential role in allowing the virus to bind to and enter the host cell. It enables the virus to attach to host cells via receptor recognition and triggers a cascade of events contributing to its pathogenesis. The S protein has vital functions that make it a target in developing a vaccine. Since the genetic sequence for the causative virus, SARS-CoV-2, was published in early January 2020 [28], the virus has mutated and shown variance between strains isolated from different continents [29]. These mutations could be responsible for the virus’s transmissibility and virulence, allowing it to adapt to different climates. Hence, a better understanding of the virus strains and their virulence can help develop an effective vaccine to fight against the pandemic. As of January 2021, the WHO has reported several vaccine candidates which consist of inactivated virus vaccines, a protein subunit vaccine, mRNA vaccines, and viral vector recombinant vaccines currently in Phase III trials [30] (Table 1).

**Table 1:** Vaccine candidates in Phase III trials as of 30 January 2021.

Manufacturer	Vaccine Candidates	Platform	Identifier	Location of Phase III Trials
Sinovac Biotech	CoronaVac	Inactivated virus	NCT04456595	Brazil
			NCT04582344	Turkey
			NCT04508075	Indonesia
			NCT04617483	China
			NCT04651790	Chile
Beijing Institute of Biological Products/Sinopharm	BBIBP-CorV	Inactivated virus (Vero cell)	NCT04560881	Argentina
			NCT04510207	United Arab Emirates, Bahrain, Egypt, Jordan
Wuhan Institute of Biological Products/Sinopharm	N/A	Inactivated virus (Vero cell)	ChiCTR2000039000	Morocco
			ChiCTR2000034780	Bahrain, Egypt, United Arab Emirates
Institute of Medical Biology/Chinese Academy of Medical Sciences	N/A	Inactivated virus (Vero cell)	NCT04659239	Brazil, Malaysia
Research Institute for Biological Safety Problems, Rep of Kazakhstan	QazCovid-in^®^-vaccine	Inactivated virus	NCT04691908	Kazakhstan
Bharat Biotech International Limited	Covaxin (BBV152B)	Inactivated virus	NCT04641481	India
			CTRI/2020/11/028976	India
Novavax	NVX-CoV2373	Protein subunit (wild-type SARS-CoV-2 spike glycoprotein)	NCT04611802	United States of America
			NCT04583995	United Kingdom
			EudraCT 2020-004123-16	United Kingdom
BioNTech/Pfizer/Fosun Pharma	Comirnaty (BNT162b2)	mRNA (nucleoside-modified RNA (modRNA) encoding the SARS-CoV-2 full-length spike, with two proline mutations to lock it in the prefusion conformation)	NCT04368728	United States of America, Argentina, Brazil, Germany, South Africa, Turkey
			NCT04713553	(Not recruiting yet)
Moderna/NIAID	mRNA-1273	mRNA (encodes SARS-CoV-2 prefusion-stabilized full-length spike protein)	NCT04649151 NCT04470427	United States of America
CureVac AG	CVnCoV	mRNA (encodes full-length spike protein of SARS-CoV-2)	NCT04674189	Germany
AstraZeneca/University of Oxford	ChAdOx1 nCoV-19/CoviShield	Viral vector (replication-deficient chimpanzee adenovirus vector the S protein of SARS-CoV-2)	NCT04400838	United Kingdom
			NCT04536051	Brazil
			ISRCTN89951424	Brazil
CanSino Biologics	Ad5-nCoV	Viral vector (adenovirus type-5 (Ad5) vector containing the S protein of SARS-CoV-2)	NCT04526990	Argentina, Chile, Mexico, Pakistan, Russia
			NCT04540419	Russia
Gamaleya Research Institute	Sputnik V (Gam-COVID-Vac)	Viral vector (recombinant adenovirus type 26 (rAd26) and recombinant adenovirus type 5 (rAd5) vectors the S protein of SARS-CoV-2)	NCT04564716	Belarus
			NCT04530396	Russia
			NCT04656613	United Arab Emirates
			NCT04642339	Venezuela
			NCT04640233	India
Janssen Pharmaceutical Companies	Ad26.COV2.S	Viral vector (recombinant, replication-incompetent adenovirus serotype 26 (Ad26) vector the S protein of SARS-CoV-2)	NCT04614948	United States of America, Belgium, France, Colombia, Germany, Philippines, South Africa, Spain, United Kingdom
			NCT04505722	United States of America, Argentina, Brazil, Chile, Colombia, Mexico, Peru, South Africa

All data collected from various clinical studies [31,32,33,34,35,36], clinical registries of China [37], US National Library of Medicine [38], International Standard Randomized Controlled Trial Number (ISRCTN) [39], and the European Union [40]. N/A: not applicable.

### 2.1. Inactivated Viral Vaccines

In an inactivated viral vaccine, the target virus has lost the ability to replicate and, thus, it is safe for use in a vaccine to trigger an immune response [41]. Inactivated vaccines predominantly trigger humoral responses that release neutralizing antibodies [42]. The inactivated viruses cannot penetrate the host cells actively; they can only rely on endocytosis to be taken up into the cells. If they are phagocytosed by dendritic cells, cross-presentation of peptides can only activate a limited number of cytotoxic T cells. Inactivated viral vaccines exhibit lesser immunogenicity and have a shorter duration of protection than live vaccines [41]. Therefore, repeat doses or co-administration with an adjuvant or delivery vehicle are needed to overcome weak immune responses after vaccination [41,43]. The addition of an adjuvant enhances local inflammation by activating the innate immune response, which increases the vaccine’s ability to provoke an adaptive response.

Among the developers of the candidate vaccines, Sinovac and Sinopharm are currently running clinical trials using inactivated vaccines. According to Sinovac, their vaccine candidate, CoronaVac, was recently approved for Phase I/II clinical trial in adolescents and children from ages 3–17 years [44]. If the results show that the vaccine is safe and effective, it could potentially be the first vaccine among the candidates that can be given to children and adolescents. In adults, during Phase I/II clinical trials for CoronaVac, the vaccine was well tolerated at different dosage levels, and no significant vaccine-related adverse events were reported. They achieved a seroconversion rate greater than 90% among the adult and elderly test subjects, indicating favorable safety and immunogenicity of the vaccine [45,46]. The vaccines from the Institute of Medical Biology and Chinese Academy of Medical Sciences and Sinovac (CoronaVac) are currently under Phase III clinical trials in Brazil, Turkey, Indonesia, China, and Chile. The trial for the CoronaVac vaccine in Brazil will involve 9000 volunteers, and the national production will commence if the vaccine is proven effective and safe [47]. Sinovac has agreed to produce at least 40 million doses of CoronaVac in Indonesia before March 2021 and will continue to supply the vaccine’s required quantity until the end of 2021 [48].

Meanwhile, the China National Pharmaceutical Group (Sinopharm) has also launched Phase III clinical trials for two candidate vaccines based on the inactivated virus. The vaccines are under development by the Wuhan Institute of Biological Products and the Beijing Institute of Biological Products. The Wuhan Institute version of the vaccine has been administered to more than 31,000 people in the United Arab Emirates (UAE), Egypt, Bahrain, and Jordan [49]. The early results show that the vaccine is safe, and there is a rise in antibodies for all the volunteers. The UAE has given emergency approval for the vaccine to be given to healthcare workers. As of January 2021, the inactivated vaccine developed by the Wuhan Institute was approved for use in the UAE and China. Currently, the Beijing Institute is working on its version of the vaccine known as BBIBP-CorV, with the Chinese Center for Disease Control and Prevention for Phase III trials [50]. The manufacturer, Sinopharm, declared that the company might produce more than one billion doses of vaccines in 2021 [51]. In late December 2020, Sinopharm announced that its vaccine, BBIBP-CorV, has an efficacy of 79.34%, thus the Chinese government has approved of its use [52,53]. In the UAE and Bahrain, the vaccine was approved for use following the announcement of a 86% efficacy rate from interim results of the Phase III clinical trials [54]. In January 2021, BBIBP-CorV was also approved in Egypt for emergency use and the vaccine will be first distributed to medical staff [55].

In India, Bahrat Biotech’s vaccine, Covaxin (BBV152), was well tolerated by the participants in Phase I/II trials. Covaxin was given in a two-dose regimen over 3 to 4 weeks and was able to induce robust humoral immune responses in all the participants. Four days after the first vaccination, the rapid humoral responses were documented. All participants achieved 100% seroconversion 42 days post immunization. Throughout the clinical trials, reported adverse events were mild to moderate. According to Bharat Biotech, Phase III clinical trials are under way, and presently the second dose of the vaccine is being distributed to the participants. The efficacy of the vaccine will be estimated by the incidence of COVID-19 cases between the vaccine and the placebo group. This will be determined two weeks after the second dose is given to the participants. It estimates that the interim results for the efficacy of the vaccine will be generated by the end of February 2021 [56,57,58]. During the pandemic, over 10 million people in India were infected by COVID-19 and over 150,000 have died because of it. Thus, there is a dire need for a safe and effective vaccine to prevent further increases in COVID-19 cases and mortalities. Covishield, AstraZeneca’s vaccine, and Covaxin were then approved for use in India in early January, and the authorities plan to vaccinate 300 million people by July 2021. Healthcare workers, emergency services, and the clinically vulnerable due to age or pre-existing comorbidities are prioritized in receiving the vaccine [58].

### 2.2. Protein Subunit Vaccines

Subunit vaccines utilize smaller components of the target pathogen to minimize the risk of unintended allergenic or reactogenic effects caused by introducing the whole organism into the host [59]. The protein subunit vaccine currently under development to fight against COVID-19 utilizes the S protein of the SARS-CoV-2 virus as it can trigger the release of neutralizing antibodies and T cells [60]. Similar to killed vaccines, subunit vaccines are not alive and so cannot penetrate host cells. Thus, the vaccine epitopes are inefficiently presented on MHC class I and can activate cytotoxic T cells only by cross-presentation, resulting in a relatively weak cytotoxic T cell response [42]. NVX-CoV2373 is a vaccine candidate currently in Phase III clinical trials designed by Novavax to protect COVID-19. It has utilized the coronavirus S protein to generate an antigen using recombinant nanoparticle technology coupled with Novavax’s Matrix-MTM adjuvant to produce their vaccine. Preliminary studies for the vaccine produced promising results whereby it demonstrated high immunogenicity in animal models [61]. Phase I/II clinical trials began in late May 2020 which included 131 participants. Only three groups out of five, namely, groups C, D, and E, were given 5 μg doses of rSARS-CoV-2 plus Matrix-M1, 25 μg doses of rSARS-CoV-2 plus Matrix-M1, and 25 μg doses of rSARS-CoV-2 plus Matrix-M1 followed by a single dose of placebo, respectively. High levels of S protein-specific antibodies and SARS-CoV-2 wild-type virus-neutralizing bodies were detected after a single immunization. This was shown on day 21 whereby the ELISA anti-spike IgG geometric mean ELISA units (GMEUs) which ranged from 105 to 116 on day 0 had risen to 1984, 2626, and 3317 GMEUs for groups C, D, and E. Upon administering the second dose with adjuvant, the microneutralization titers further increased 8-fold to 15, 319 and 20, 429 GMEUs over responses seen with the first vaccination in groups C and D [32]. Neutralization titers are correlated with protection against viral infections post vaccination. Vaccines against viral infections induce protective neutralizing antibodies which are markers of protective immunity against reinfections after an initial infection [62]. This led the team to believe that the vaccine would be protective when used in humans. The results have shown that the vaccine, which consists of the viral antigen and adjuvant, was generally well tolerated and was able to elicit robust antibody responses. Novavax launched Phase III trials in September with 10,000 volunteers in the United Kingdom and developed Phase III trials at a larger scale in the United States in October 2020 [63,64]. They could potentially be delivering results in early 2021. If the trials succeed, Novavax is expected to have 100 million doses of vaccines in the United States and to produce 2 billion doses a year as per their agreement with a major vaccine manufacturer, the Serum Institute of India.

### 2.3. mRNA Vaccines

The mRNA vaccines are categorized under nucleic acid vaccines, whereby they deliver genetic codes to the host cell ribosomes to stimulate viral protein synthesis. Exogenous mRNA from the vaccine can stimulate an immune response as innate immune receptors recognize it on the cell surface, endosomal, and cytosolic innate immune receptors [65]. Once the innate immune system is triggered, chemokines and cytokines essential for an effective adaptive immune response to be induced are released [65]. As the encoded viral protein is synthesized, the immune system will recognize the protein and elicit the desired immune response [66,67]. The proteins will be presented by antigen-presenting cells, causing a cascade of events, including T cell proliferation, immune cell activation, and activating memory B cells for adaptive immunity [65]. Since mRNA only has to be in the cytosol via membrane-derived endocytic pathways [68] to be translated by the ribosomes, they can avoid integration into the host genome. It has also been found that mRNA vaccines allow transient expression and accumulation of the target viral antigens [69,70], thus creating a profound effect on the magnitude and affinity maturation of antibody responses, providing a longer duration of protection [71]. Besides, mRNA vaccines are non-infectious and non-integrating, which means a significantly lower risk of infection or insertional mutagenesis [72]. Furthermore, mRNA is degraded in the body through normal cellular processes. Thus, the current vaccine candidate developers, Moderna and BioNTech, both use intramuscular injections to administer their vaccine. This prevents rapid digestion of the mRNA by ribonucleases and avoids stimulation of the innate immune response that could reduce the vaccine’s efficacy [73].

In January 2020, the team at Moderna was able to finalize the sequence for mRNA-1273 and managed to produce the first clinical batch of mRNA-1273 in 25 days from sequence selection. The vaccine candidate encodes the S-2P antigen, made up of the SARS-CoV-2 glycoprotein with a transmembrane anchor and an intact S1-S2 cleavage site. The antigen is then stabilized in its prefusion conformation via the substitution of amino acids with proline. The lipid nanoparticle capsule containing four lipids was formulated in a fixed ratio of mRNA and lipid [74]. A Phase I study commenced in March, and the results showed that the vaccine was able to induce anti-SARS-CoV-2 immune responses in all participants [74]. No serious adverse events were found; thus, the study could proceed to Phase II in late May and, subsequently, Phase III studies in late July. Phase III studies have since enrolled 30,000 healthy people from across the United States at approximately 89 sites to participate in the trials. The US government has also awarded the company an additional USD 1.5 billion in exchange for 100 million doses if the vaccine is proven safe and effective. In mid-September, Moderna announced the protocol which it uses to determine the safety and efficacy of the vaccine. It planned to wait until a significant number of volunteers became sick with COVID-19 and then see how many had been vaccinated. In a press conference held in July, the president of Moderna stated that it found that the vaccine protects non-human primates from SARS-CoV-2. At both 10 µg and 100 µg dose levels, mRNA-1273 was found to protect against lung inflammation and viral replication in the lungs in non-human primates challenged by SARS-CoV-2. Animals that were given 100µg of the vaccine demonstrated protection against viral replication in their noses. These results, coupled with the now published Phase I clinical data for the vaccine, both demonstrate similar protective immune responses. Moderna was optimistic that mRNA-1273 will be able to prevent COVID-19 disease and slow down the spread of the virus [75]. Subsequently, in the Phase III clinical trials, mRNA-1273 was shown to have 94.1% efficacy in preventing COVID-19 illnesses in the participants with transient adverse reactions [76]. This then led to the authorization of Moderna’s vaccine for emergency use in the United States of America and the United Kingdom and it has also been distributed for use in numerous other countries [77].

Similarly, BioNTech, in collaboration with Pfizer and Fosun Pharma, are also currently developing an mRNA vaccine. The vaccine is to be given in two doses. They launched Phase I/II trials with two versions of the vaccine and found that both versions caused volunteers to produce anti-SARS-CoV-2 antibodies as well as immune T cells that responded to the virus. Among the two versions, BNT162b2 caused significantly fewer side effects such as fatigue and fevers; thus, it was chosen to move on to Phase II/III trials. The trials enrolled 30,000 volunteers from the United States, Argentina, Brazil, and Germany. Volunteers reported that they experienced mild to moderate side effects after receiving the first dose. Pfizer and BioNTech then sought expansion of the trials, and they are the first American trials to have gained permission to begin testing the vaccine on children. In an open letter from the chairman of Pfizer, he stated, “Pfizer will apply for Emergency Authorization Use in the US soon after the safety milestone is achieved in the third week of November” [78]. In early November, BioNTech and Pfizer announced that their vaccine demonstrated 90% effectiveness in preventing COVID-19 infections in the first set of results from their ongoing Phase III trials. The vaccine prevented COVID-19 infections in participants with no previous evidence of SARS-CoV-2 infections without causing severe adverse events [79]. In December 2020, Pfizer released a statement summarizing their Phase III clinical results, stating that the vaccine was 95% effective against COVID-19 disease and was well tolerated among the participants [31]. Thus, Pfizer was able to be the first vaccine developer to obtain authorization for emergency use for their vaccine, BNT162b2, in the United States of America. Other countries, such as the United Kingdom and Canada, have also approved its emergency use [80]. The Pfizer–BioNTech vaccine has since been distributed to countries all around the world to help fight against the pandemic. The team expects to manufacture over 1.3 billion doses of their vaccine worldwide by the end of 2021. Getting the vaccine from the factory to people’s arms could pose some significant challenges. Like Moderna’s, Pfizer and BioNTech’s preparation is based on mRNA, which falls apart unless the vaccine is transported and stored frozen. As a result, the vaccine will have to be chilled to minus 80 degrees Celsius until it is ready to be injected.

### 2.4. Recombinant Viral Vector Vaccines

Recombinant viral vector vaccines are generally built upon a non-replicative virus or an attenuated replication-competent virus, which is bioengineered to express antigens from the target pathogen. Although various viral vectors are being studied in vaccine development [81,82], only a few recombinant viral vector vaccines have been approved for human use. Replication-defective adenoviruses are the vectors of choice for COVID-19 vaccine development. The adenoviral vectors can enter the host cells to replicate and produce viral proteins via gene transcription. The synthesized viral proteins can then induce potent antibody and T cell responses [83]. Adenoviruses are a suitable vaccine vector as they fit the criteria of high transduction efficiency, high level of transgene expression, and a broad range of viral tropism. They can infect both dividing and non-dividing cells. Utilizing adenoviruses allows for a smaller dose necessary to induce an immune response that lasts longer. However, pre-existing immunity in the host is an issue that hinders the vaccine’s efficacy [84,85,86,87].

Four vaccine candidates using the recombinant viral vector platform have commenced Phase III trials in several countries. CanSino Biological Inc. has collaborated with the Beijing Institute of Biotechnology to develop a non-replicating adenovirus type-5 (Ad5)-vectored COVID-19 vaccine named Ad5-nCoV. In March 2020, results from their Phase I trial provided evidence that the Ad5-vectored COVID-19 vaccine is tolerable and immunogenic at 28 days post vaccination. Rapid specific T cell responses were also noted from 14 days post immunization, and humoral responses peaked 28 days post vaccination. Reported adverse events were mild to moderate, and no significant severe adverse reactions were reported [88]. Phase II trials in April 2020 found that the vaccine was safe when the volunteers were dosed at 5 × 10^10^ viral particles. Only one participant out of 129 experienced severe adverse reactions. Although mild to moderate adverse reactions did occur post vaccination, the symptoms resolved within 48 h. The vaccine was also able to induce significant immune responses in most recipients after a single immunization [34]. The vaccine was then approved for military use in China in June 2020 [89]. Phase III trials of Ad5-nCoV are being done in Pakistan, Russia, and Saudi Arabia [45]. Phase II and Phase III clinical trials of Ad5-nCoV showed that the vaccine was effective and safe for use without causing severe adverse events [45].

The vaccine candidate currently being developed at the University of Oxford in collaboration with AstraZeneca, named ChAdOx1 nCoV-19 (AZD1222), is based on a chimpanzee adenovirus. The use of ChAdOx1 as the viral backbone could help avoid pre-existing immunity in the host cells. Phase I/II trials carried out between April and May 2020 found that the vaccine has an acceptable safety profile. Furthermore, upon administering a booster dose, neutralizing antibody responses increased. This vaccine, like the other candidates, was also able to induce humoral and cellular immune responses. No severe adverse reactions were reported, and only mild to moderate reactions were reported [90]. Subsequently, Phase II/III trials began in England and India, where the vaccine was known as Covishield. At the same time, AstraZeneca launched Phase III trials in Brazil, South Africa, and the United States. The company has also agreed to supply 400 million doses to the European Union if the vaccine is proved to be safe and effective [91]. However, global trials were put on hold momentarily because one volunteer was diagnosed with transverse myelitis, an inflammatory syndrome that affects the spinal cord caused by viral infections [92]. Upon review, it was ruled that the vaccine was not the cause of the inflammatory syndrome. Another volunteer from Brazil died after participating in the trials, but whether the man received the experimental vaccine or placebo remains undisclosed. AstraZeneca resumed its trials since not enough evidence has been able to confirm the correlation between the events and the vaccine. Upon completion of the Phase III trials, AZD1222 had a 70.4% efficacy against COVID-19 infection after two doses and an efficacy of 64.1% against symptomatic disease after one standard dose [33]. As it has proven to be effective and has an acceptable safety profile, AZD1222 became the second vaccine to be approved in the UK and will be given to prioritized groups first [93]. The vaccine was also later approved for use in Europe and India [58,91].

Johnson & Johnson has developed their vaccine candidate using Ad26 to fight against COVID-19. It has previously used recombinant adenovirus serotype 26 (Ad26) to create a vaccine for Ebola. Phase I/II trials that began in July yielded results that stated the vaccine had a good safety profile and immunogenicity after only a single dose [36]. The vaccine induced an immune response in the participants with a high seroconversion rate, and only mild to moderate side effects were reported [94]. This led to Phase III trials with 60,000 participants in September. The company declared on their company website that Johnson & Johnson will supply 100 million doses to the United States’ federal government for USD 1 billion [95] and supply 200 million doses to the European Union members following approval or authorization from the regulators [96]. On October 12, the company announced that it would pause their clinical trials due to a participant experiencing an unexplained illness. It halted the trials to investigate the reaction’s nature to determine whether it was related to the vaccine given [97]. Nevertheless, 11 days after the report, Johnson & Johnson resumed the trials after concluding that the medical event had no clear indication that it was related to the vaccine. This is because nobody knew if the vaccine or placebo was given to the patient [89]. Towards the end of January 2021, Johnson & Johnson announced that their Ad26.COV2.S vaccine was 66% effective in preventing moderate to severe COVD-19, 28 days after vaccination. The first onset of protection was observed on day 14 post immunization. This single-shot vaccine was reported to be safe for use as no significant safety concerns occurred during the trials. The company has plans to file for US emergency use authorization (EUA) and upon receiving the approval, it expects to have product available for shipment [95].

In Russia, the Gamaleya Research Institute is currently developing a vaccine candidate named Sputnik V (GAM-COVID-Vac), a combination of Ad5 and Ad26, each engineered with a coronavirus gene. The purpose of using two different adenoviruses is to increase the effectiveness of the vaccine [98,99]. Phase I trials showed that both formulations were well tolerated and only mild to moderate adverse effects were detected. All participants produced antibodies to SARS-CoV-2 glycoprotein. The vaccine induced strong humoral and cellular immune responses with a good safety profile [35]. The institute has also stated that none of the participants were infected with COVID-19 after administering the vaccine [100]. The vaccine had been approved for use under a “conditional registration certificate,” which would depend on positive results from Phase III trials. Phase III trials expanded to 40,000 volunteers recruited from Russia, Belarus, and the United Arab Emirates, while Phase II/III trials were launched in India. Although clinical trials for this vaccine were still ongoing in December 2020, the manufacturer declared that Sputnik V has been registered by the Ministry of Health of the Republic of Belarus, making it the first country to do so outside Russia [101]. The Phase III trials in Russia were reported in February 2021, stating that the vaccine was 91.6% efficacious against infection. The onset of protection is 21 days after the first dose, the same day of receiving the second dose. Overall, the vaccine was well tolerated in the participants, with only 0.3% (45) participants experiencing severe adverse events. Although deaths were reported in the study, none of them could be attributed to Sputnik V as the cause of death was due to pre-existing conditions in the participant. GAM-COVID-Vac has already been distributed for public use in Russia, with the majority given to at-risk populations, medical workers, and teachers. Over 2 million doses of the locally produced vaccine have been distributed in Russia since 23 January 2021 [100,102].

## 3. Vaccines Currently in Use

On 18 November 2020, Pfizer announced that their vaccine candidate, BNT162b2, has a 95% efficacy against COVID-19 among participants of various ages, genders, races, and ethnicities (Table 2) [31]. In participants over 65 years of age, the vaccine was over 94% efficient against the virus. The results of the final efficacy analysis of Pfizer’s Phase III study of the vaccine candidate led Pfizer to achieve the safety milestone required by the US Food and Drug Administration (FDA) for emergency use authorization (EUA) [103]. The Pfizer–BioNTech COVID-19 vaccine is an mRNA vaccine with synthetic and enzymatically produced components. It does not contain any live virus; thus, upon administration, the recipient will not develop COVID-19. The vaccine works by introducing a small portion of the gene sequence of SARS-CoV-2 into the recipient, whereby the host mechanisms will then produce spike proteins of the virus. The immune system will then recognize the viral spike proteins and elicit an immune response, which subsequently builds up immunological memory. This allows the host to gain immunity for fighting against future infection. The vaccine has been approved by the FDA and authorized for emergency use [104]. The vaccine has since been distributed in numerous countries such as the United States of America, the United Kingdom, Belgium, Canada, Costa Rica, the Czech Republic, Greece, Hungary, Israel, Kuwait, Mexico, Oman, Poland, Qatar, Saudi Arabia, Serbia, Slovakia, Switzerland, and the United Arab Emirates. The first rollout of the vaccine was given to frontline healthcare workers and the elderly who are more susceptible to the infection.

It is currently available to be administered via the intramuscular route to individuals 16 years old and older. Two doses of 30mcg/0.3 mL each will be given to the individuals 3 weeks apart. They are advised to visit the nearest healthcare practitioner or hospital to seek proper management of side effects should they occur. It is contraindicated to use this vaccine in individuals who have previously experienced allergic reactions to any vaccine component. For immunosuppressed individuals, extra caution and care are needed during vaccination as they may have a reduced immune response towards the vaccine. Thus, it is advised that the use of the vaccine in immunocompromised individuals would require close monitoring, and treatment for severe allergic reactions should be available immediately upon vaccination [105]. Upon receiving the vaccine, it typically takes the human body a few weeks to produce antibodies. Therefore, if an individual were exposed to the virus right before or after the immunization, they could still be susceptible to the infection as there would be insufficient time for the vaccine to elicit antibodies for protection [106]. After receiving the second dose, the vaccine may take approximately 7 to 10 days to provide protection. Thus, it is important to receive the second dose three weeks after the first vaccine is administered [107]. There are no conclusive data on the duration of the protection that this vaccine will provide nor whether a booster dose is required. Thus, Pfizer will follow up with the clinical trial participants for up to 24 months post vaccination to determine the duration of action of the vaccine [108].

The vaccine by Moderna, mRNA-1273, was reported to have a 94.5% effectiveness in preventing COVID-19 disease as 95 confirmed cases were documented in the company’s trial of 30,000 participants [109]. Similarly to Pfizer’s vaccine, the FDA has not approved Moderna’s vaccine, but it was the second vaccine authorized for emergency use in the US [77]. In the United Kingdom, Moderna’s vaccine was approved as the third coronavirus vaccine for emergency use by the UK’s medicine regulator. It is now expected to deliver 17 million doses to the country [110]. The vaccine was also approved in Europe in early January to help fight against the pandemic [111]. On 20 December 2020, Moderna began transporting its vaccine to healthcare facilities throughout the United States of America [107]. The Moderna vaccine is encapsulated in lipid nanoparticles, and it consists of the mRNA responsible for the production of SARS-CoV-2 spike proteins. With similar mechanisms as the Pfizer vaccine, Moderna’s vaccine introduces the mRNA to the host’s body, triggering the expression of the viral spike protein, which will induce an immune response. This vaccine is given in two doses of 100 mcg/0.5 mL and the doses are spaced 28 days apart. The dosing interval can be extended to 42 days if necessary [76]. The vaccine can be given via an injection into the muscle of individuals 18 years old and over. Individuals who have previously had a severe allergic reaction to vaccine components should not take the vaccine [75]. In addition, immunosuppressed patients can also receive this vaccine if they have no contraindications to this vaccination. These individuals should be notified of the side effects and the safety profile of the vaccine. Due to their reduced immune response, it is important to ensure immediate medical treatment can be given if a severe allergic reaction occurs post immunization [112,113]. Moderna’s vaccine has been shown to produce a protective effect 14 days after the first dose is given. As already mentioned, vaccines can take a certain time to produce protection for the recipient, thus individuals need to ensure they receive the second dose of the vaccine [76]. However, a study reported that the duration of protective effects of Moderna’s vaccine could potentially be less than a year in elderly individuals. In the study, individuals over 56 years old demonstrated a decrease in neutralizing antibody counts to anywhere between 50 and 75 percent three months after receiving the second dose of vaccine. Individuals within these age groups are most susceptible to the infection, thus this could be a potential issue as the duration of action of the vaccine is possibly shorter than expected [114]. Nevertheless, the study only included 34 participants, therefore further monitoring of antibody counts in individuals at a greater scale should be conducted to obtain conclusive data.

AstraZeneca has also developed a vaccine against SARS-CoV-2 in collaboration with Oxford, named ChAdOx1 nCoV-19 (AZD1222). The vaccine was documented to have a 70.4% efficacy against the viral infection after two doses. Moreover, the vaccine provided protection at an efficacy of 64.1% against symptomatic disease after one standard dose [33]. However, in comparison with Pfizer and Moderna’s vaccines, the efficacy is significantly lower. At the end of December 2020, Oxford–AstraZeneca’s vaccine was approved for use in the UK [93]. In the United Kingdom, the first recipients of the vaccine will be frontline healthcare workers, care home residents and workers, the elderly, and individuals who have other comorbidities [93]. This vaccine works on a recombinant viral vector vaccine platform in which the genes for the spike protein on SARS-CoV-2 are inserted into a vector, an adenovirus. The vaccine is then introduced into the human body via injection where the adenovirus containing the viral spike proteins can elicit an immune response. As the immune system detects these viral proteins’ presence, an immune response is triggered, which eventually builds up immunological memory in the host. If an infection occurs in the future, the immune system will be triggered and antibodies will be released quickly to fight against the disease [115]. AstraZeneca’s vaccine is administered to individuals 18 years old and over through the intramuscular route with two doses of 0.5 mL each with an interval of four to twelve weeks. The vaccine should not be given to any individual who has experienced a severe allergic reaction to the contents of a vaccine. There are no conclusive data that show that immunocompromised individuals will have the same immune response as immunocompetent individuals to the vaccine. It has been suggested by the UK’s Commission on Human Medicines that the vaccine can provide 70% protection after 21 days, and the second dose could give up to 80% protection. The second vaccination is suggested to be given 12 weeks after the first dose [116].

### Side Effects of the Current Vaccines in Use

Multiple studies have been done to determine the safety, tolerability, and immunogenicity of COVID-19 vaccines. Although, in general, all vaccines can induce an immune response to release neutralizing and IgG antibodies to fight against the viral protein, adverse events could also potentially occur post vaccination. The common side effects (pain or swelling at the injection site, mild fever, chills, fatigue, headache, and muscle aches) are mostly mild to moderate in nature and subside within a few days [117,118]. Following the administration of Pfizer–BioNTech’s vaccine to the general public outside of clinical trials, adverse reactions have been reported [119]. The most reported side effects were generally mild, ranging from injection site soreness, fatigue, headache, myalgia, chills, joint pain, fever, and nausea. These symptoms were also present in some of the participants during the clinical trials, and severe allergic reactions were reported in rare instances. Similar side effects have also been reported for Moderna and AstraZeneca’s vaccine [52,119]. However, after the distribution and administration of the Pfizer–BioNTech vaccine, thirty-three deaths were reported in mid-January 2021. These deaths occurred among 42,000 people who were given the vaccination. Upon review, the deceased were all over 75 years of age and some were on the verge of death as they were terminally ill. Reports from Germany state that at least seven older adults have died post vaccination, but their deaths could be attributed to their underlying chronic medical conditions and were not due to vaccination [119]. The Centers for Disease Control and Prevention (CDC) has reported 45 cases of anaphylaxis with the Pfizer vaccine, which can be translated to 6.2 cases per million doses, while 19 cases have been reported for Moderna’s vaccine, which is translated to 2.1 cases per million doses [120]. This further indicates that the risk of anaphylaxis is relatively low with these vaccines. As anaphylactic reactions typically occur within minutes to hours after vaccination, healthcare workers in the US observe recipients of the vaccine for at least 15 min after immunization. Individuals who have a concerning history of allergic reactions are monitored for a more extended period after vaccination [120,121]. Additionally, Moderna’s vaccine was reported to only elicit side effects in 1266 (0.03%) among over 4 million people who received the first dose of the vaccine [122]. As for the vectored vaccine by AstraZeneca, a participant in its clinical trials suffered from inflammation of the spinal cord after receiving the vaccine. This led to the halting of clinical trials until experts determined the cause of the inflammation to be unrelated to the vaccine. In the clinical trials, local and systemic reactions such as pain at the injection site, myalgia, headache, fever, and fatigue were documented [123]. Further data on the adverse reactions or side effects caused by Oxford–AstraZeneca’s vaccine are needed to determine its safety and efficacy. The first vaccine was only recently administered in early January 2021.

## 4. Conclusions

The different vaccine platforms’ basic concepts have been thoroughly studied, and each comes with benefits and limitations. A summary of the advantages and disadvantages of the various vaccine platforms is illustrated in Table 3. To overcome the vaccine platforms’ limitations, various methods to modify the vaccine platforms have been developed to ensure the vaccines are safe and efficacious before being distributed. With inactivated viral vaccines and protein subunit vaccines, the antigens in the vaccine alone may have insufficient immunostimulatory capabilities; thus, the addition of adjuvants is needed. Adjuvants mainly trigger the innate immune response to recognize the vaccine components as a threat to the host, which then activates the antigen-presenting cells, resulting in adaptive immune activities [124]. This increases the efficacy of the vaccine, along with prolonging the duration of the vaccine. It could also possibly mean that a lower dosing frequency is needed to achieve immunity towards the pathogen. However, an adjuvant may increase local reactions such as pain, redness, and swelling at the injection site and a few systemic symptoms such as fever, fatigue, and myalgia [125]. This could be due to enhanced activation of the immune response, resulting in inflammation at the injection site. Currently, the mRNA vaccines developed by Pfizer and Moderna do not utilize adjuvants. Prime-boost strategies have also been used to develop higher immunity levels compared to immune responses obtained by a single vaccination [126]. This approach aims to induce both humoral and cellular immune responses to produce long-lasting immunity. An example would be using a heterologous prime-boost method involving a viral vector for priming a protein-based vaccine as the booster. This strategy allows for the induction of a strong cellular immune response and a higher specificity of antibodies produced against the vaccine target [127].

Studies have shown that modifications applied to vaccines could potentially cause other side effects even though they may increase the vaccine’s effectiveness. There have been instances where licensed vaccines have been withdrawn from the market due to newly discovered adverse events associated with the vaccine. Thus, assessment of the benefit–risk ratio is essential to ensure that the continued use of the vaccine brings more benefits than harm to the public. As the disease comes under control or new information regarding the vaccine arises, evaluations of the benefits of the vaccines may change. As all the Phase III trials are still ongoing for the COVID-19 vaccines, it is still difficult to ascertain which vaccine candidate will be the most suitable and effective in fighting against the COVID-19 pandemic. Each vaccine candidate has demonstrated that it can elicit an immune response and has a relatively good safety profile. All the vaccine platforms used have unique advantages and disadvantages. The most suitable vaccine choice ultimately depends on the knowledge of the pathophysiology of SARS-CoV-2 and the immune response required for protection. Regardless, further evidence from the clinical trials is still needed to confirm that the vaccine can be used without causing severe complications in the host post immunization. COVAX, launched in April by the WHO, is a global collaboration that supports the research, development, manufacturing, and price negotiation of the vaccine candidates. COVAX has been established to accelerate the successful development of a vaccine for COVID-19 and initiatives have been implemented to ensure that nations with lower incomes will also be able to receive the vaccines. However, the New York Times reported that richer countries such as Canada, the United States of America, and the United Kingdom have purchased enough doses of vaccines to vaccinate their nations multiple times over [128,129]. As the richer countries place larger orders, the ability of other countries to procure the vaccines could be severely undermined and could lead to more deaths globally due to the coronavirus. The promising results from the clinical trials have led researchers to be hopeful and optimistic that a vaccine for COVID-19 will be on the market in 2021 to put an end to the pandemic.

Recently, the United Kingdom approved the use of vaccines produced by Pfizer and BioNTech [80], and other countries worldwide are following in its footsteps. In the Middle East, three countries, Israel, the United Arab Emirates (UAE), and Bahrain, have successfully administered the vaccine to a high percentage of their populations compared to the rest of the world. These Middle Eastern states have put continuous effort into reassuring the public that the vaccine is safe and effective. Through its universal healthcare system, within days, Israel vaccinated almost 11.0 doses per 100 population and the next highest rate was 3.5 in Bahrain. The vaccination in these countries was higher compared to that in the United Kingdom, the United States of America, and even China [130] Researchers are hopeful that once a greater percentage of the general population receives the vaccine, herd immunity can be achieved, and we can finally put an end to this pandemic. Further surveillance on the long-term effects of COVID-19 infections can help healthcare professionals to better treat the disease. Continuous monitoring of recipients of the vaccine is also essential to detect any severe adverse reactions to ensure the vaccine is safe for use and future approval. Vaccination is the prime solution now, however, we ought to be careful about its side effects and the occurrence of mutant COVID-19 strains.

## Figures and Tables

**Table 2:** The differences between the vaccines currently in use.

Manufacturer	Vaccine	Platform	Differences
Pfizer–BioNTech	BNT162b2	mRNA	95% efficacy Available for 16-year-olds and over Doses spaced three weeks apart Protection in 7 to 10 days after second dose
Moderna	mRNA-1273	mRNA	94.1% efficacy Available for 18-year-olds and over Doses spaced four weeks apart Protection in 14 days after first dose
AstraZeneca	ChAdOx1 nCoV-19 (AZD1222)	Viral vector	70% efficacy Available for 18-year-olds and over Doses spaced four to twelve weeks apart Protection in 21 days after first dose

**Table 3:** Advantages and disadvantages of various vaccine platforms.

Vaccine Platform	Advantage	Disadvantage
Inactivated virus	Good safety profile Better stability during transportation and storage Longer shelf life	Weak immunogenicity Shorter duration of protection Requires adjuvants Large dose required
Protein subunit	Non-infectious Fewer side effects	Limited immunogenicity Require adjuvants
mRNA	Non-infectious Non-integrating High malleability to regulate immunogenicity High immunogenicity Long-lasting immunity	Stability *in-vivo*
Recombinant viral vector	Unwanted antigens can be eliminated Adjuvants not required due to danger signals from vector High efficiency in stimulating immune responses	Pre-existing host immunity reduces efficacy Costly

## References

[1] Shereen M.A., Khan S., Kazmi A., Bashir N., Siddique R. (2020). COVID-19 infection: Origin, transmission, and characteristics of human coronaviruses. J. Adv. Res..

[2] WHO Timeline: WHO’s COVID-19 Response. https://www.who.int/emergencies/diseases/novel-coronavirus-2019/interactive-timeline#event-7.

[3] Dong E., Du H., Gardner L. (2020). An interactive web-based dashboard to track COVID-19 in real time. Lancet Infect. Dis..

[4] Hopkins J. Coronavirus COVID-19 Global Cases. https://coronavirus.jhu.edu/map.html.

[5] Spinner C.D., Gottlieb R.L., Criner G.J., López J.R.A., Cattelan A.M., Viladomiu A.S., Ogbuagu O., Malhotra P., Mullane K.M., Castagna A. (2020). Effect of remdesivir vs standard care on clinical status at 11 days in patients with moderate COVID-19: A randomized clinical trial. JAMA.

[6] Letchumanan V., Ab Mutalib N.-S., Goh B.-H., Lee L.-H. (2020). Novel coronavirus 2019-nCoV: Could this virus become a possible global pandemic. Prog. Microbes Mol. Biol..

[7] Naqvi A.A.T., Fatima K., Mohammad T., Fatima U., Singh I.K., Singh A., Atif S.M., Hariprasad G., Hasan G.M., Hassan M.I. (2020). Insights into SARS-CoV-2 genome, structure, evolution, pathogenesis and therapies: Structural genomics approach. Biochim. Biophys. Acta Mol. Basis Dis..

[8] Huang Y., Yang C., Xu X.-f., Xu W., Liu S.-W. (2020). Structural and functional properties of SARS-CoV-2 spike protein: Potential antivirus drug development for COVID-19. Acta Pharmacol. Sin..

[9] Kaur S.P., Gupta V. (2020). COVID-19 Vaccine: A comprehensive status report. Virus Res..

[10] Astuti I. (2020). Severe Acute Respiratory Syndrome Coronavirus 2 (SARS-CoV-2): An overview of viral structure and host response. Diabetes Metab. Syndr..

[11] Galbadage T., Peterson B.M., Gunasekera R.S. (2020). Does COVID-19 Spread Through Droplets Alone?. Front. Public Health.

[12] Zheng J. (2020). SARS-CoV-2: An Emerging Coronavirus that Causes a Global Threat. Int. J. Biol. Sci..

[13] Jordan R.E., Adab P., Cheng K. (2020). Covid-19: Risk factors for severe disease and death. BMJ.

[14] Lee V.S., Chong W.L., Sukumaran S.D., Nimmanpipug P., Letchumanan V., Goh B.H., Lee L.-H., Zain S.M., Abd Rahman N. (2020). Computational screening and identifying binding interaction of anti-viral and anti-malarial drugs: Toward the potential cure for SARS-CoV-2. Prog. Drug Discov. Biomed. Sci..

[15] Zhong N., Zheng B., Li Y., Poon L., Xie Z., Chan K., Li P., Tan S., Chang Q., Xie J. (2003). Epidemiology and cause of severe acute respiratory syndrome (SARS) in Guangdong, People’s Republic of China, in February, 2003. Lancet.

[16] Rabaan A.A., Al-Ahmed S.H., Sah R., Alqumber M.A., Haque S., Patel S.K., Pathak M., Tiwari R., Yatoo M.I., Haq A.U. (2021). MERS-CoV: Epidemiology, molecular dynamics, therapeutics, and future challenges. Ann. Clin. Microbiol. Antimicrob..

[17] Lin J., Zhang J.-S., Su N., Xu J.-G., Wang N., Chen J.-T., Chen X., Liu Y.-X., Gao H., Jia Y.-P. (2007). Safety and immunogenicity from a phase I trial of inactivated severe acute respiratory syndrome coronavirus vaccine. Antivir. Ther..

[18] Martin J.E., Louder M.K., Holman L.A., Gordon I.J., Enama M.E., Larkin B.D., Andrews C.A., Vogel L., Koup R.A., Roederer M. (2008). A SARS DNA vaccine induces neutralizing antibody and cellular immune responses in healthy adults in a Phase I clinical trial. Vaccine.

[19] Modjarrad K., Roberts C.C., Mills K.T., Castellano A.R., Paolino K., Muthumani K., Reuschel E.L., Robb M.L., Racine T., Oh M.-D. (2019). Safety and immunogenicity of an anti-Middle East respiratory syndrome coronavirus DNA vaccine: A phase 1, open-label, single-arm, dose-escalation trial. Lancet Infect. Dis..

[20] Greenwood B. (2014). The contribution of vaccination to global health: Past, present and future. Philos. Trans. R. Soc. Lond. B Biol. Sci..

[21] Johnson D., Ren S.E.C., Johnson H.D., Letchumanan V. (2020). COVID-19: Are Malaysians embracing or suffering the new normality?. Prog. Microbes Mol. Biol..

[22] Ser H.-L., Letchumanan V., Law J.W.-F., Tan L.T.-H., Ab Mutalib N.-S., Lee L.-H. (2020). PMMB COVID-19 Bulletin: Spain (18th April 2020). Prog. Microbes Mol. Biol..

[23] Tan L.T.-H., Letchumanan V., Ser H.-L., Law J.W.-F., Ab Mutalib N.-S., Lee L.-H. (2020). PMMB COVID-19 Bulletin: United Kingdom (22nd April 2020). Prog. Microbes Mol. Biol..

[24] Lurie N., Saville M., Hatchett R., Halton J. (2020). Developing Covid-19 vaccines at pandemic speed. N. Engl. J. Med..

[25] Beverley P.C.L. (2002). Immunology of vaccination. Br. Med. Bull..

[26] Ada G. (1991). The ideal vaccine. World J. Microbiol. Biotechnol..

[27] Clem A.S. (2011). Fundamentals of vaccine immunology. J. Glob. Infect. Dis..

[28] Wu F., Zhao S., Yu B., Chen Y.-M., Wang W., Song Z.-G., Hu Y., Tao Z.-W., Tian J.-H., Pei Y.-Y. (2020). A new coronavirus associated with human respiratory disease in China. Nature.

[29] Ser H.-L., Tan L.T.-H., Law J.W.-F., Letchumanan V., Ab Mutalib N.-S., Lee L.-H. (2020). Genomic analysis of severe acute respiratory syndrome coronavirus 2 (SARS-CoV-2) strains isolated in Malaysia. Prog. Microbes Mol. Biol..

[30] WHO Draft Landscape and Tracker of COVID-19 Candidate Vaccines. https://www.who.int/publications/m/item/draft-landscape-of-covid-19-candidate-vaccines.

[31] Polack F.P., Thomas S.J., Kitchin N., Absalon J., Gurtman A., Lockhart S., Perez J.L., Pérez Marc G., Moreira E.D., Zerbini C. (2020). Safety and efficacy of the BNT162b2 mRNA Covid-19 vaccine. N. Engl. J. Med..

[32] Keech C., Albert G., Cho I., Robertson A., Reed P., Neal S., Plested J.S., Zhu M., Cloney-Clark S., Zhou H. (2020). Phase 1–2 trial of a SARS-CoV-2 recombinant spike protein nanoparticle vaccine. N. Engl. J. Med..

[33] Voysey M., Clemens S.A.C., Madhi S.A., Weckx L.Y., Folegatti P.M., Aley P.K., Angus B., Baillie V.L., Barnabas S.L., Bhorat Q.E. (2021). Safety and efficacy of the ChAdOx1 nCoV-19 vaccine (AZD1222) against SARS-CoV-2: An interim analysis of four randomised controlled trials in Brazil, South Africa, and the UK. Lancet.

[34] Zhu F.-C., Guan X.-H., Li Y.-H., Huang J.-Y., Jiang T., Hou L.-H., Li J.-X., Yang B.-F., Wang L., Wang W.-J. (2020). Immunogenicity and safety of a recombinant adenovirus type-5-vectored COVID-19 vaccine in healthy adults aged 18 years or older: A randomised, double-blind, placebo-controlled, phase 2 trial. Lancet.

[35] Logunov D.Y., Dolzhikova I.V., Zubkova O.V., Tukhvatullin A.I., Shcheblyakov D.V., Dzharullaeva A.S., Grousova D.M., Erokhova A.S., Kovyrshina A.V., Botikov A.G. (2020). Safety and immunogenicity of an rAd26 and rAd5 vector-based heterologous prime-boost COVID-19 vaccine in two formulations: Two open, non-randomised phase 1/2 studies from Russia. Lancet.

[36] Sadoff J., Le Gars M., Shukarev G., Heerwegh D., Truyers C., de Groot A.M., Stoop J., Tete S., Van Damme W., Leroux-Roels I. (2021). Interim Results of a Phase 1–2a Trial of Ad26. COV2. S Covid-19 Vaccine. N. Engl. J. Med..

[37] Huang J., He Y., Su Q., Yang J. (2020). Characteristics of COVID-19 Clinical Trials in China Based on the Registration Data on ChiCTR and ClinicalTrials.gov. Drug Des. Devel. Ther..

[38] U.S. National Library of Medicine-ClinicalTrials.gov. https://clinicaltrials.gov/ct2/home.

[39] ISRCTN Registry. http://www.isrctn.com/ISRCTN89951424.

[40] EU Clinical Trials Register. https://www.clinicaltrialsregister.eu/ctr-search/search.

[41] Tlaxca J.L., Ellis S., Remmele R.L. (2015). Live attenuated and inactivated viral vaccine formulation and nasal delivery: Potential and challenges. Adv. Drug Del. Rev..

[42] Mak T.W., Saunders M.E., Mak T.W., Saunders M.E., Jett B.D. (2014). Chapter 14-Vaccination. Primer to the Immune Response.

[43] Vetter V., Denizer G., Friedland L.R., Krishnan J., Shapiro M. (2018). Understanding modern-day vaccines: What you need to know. Ann. Med..

[44] Tumban E. (2021). Lead SARS-CoV-2 Candidate Vaccines: Expectations from Phase III Trials and Recommendations Post-Vaccine Approval. Viruses.

[45] Sharma O., Sultan A.A., Ding H., Triggle C.R. (2020). A Review of the Progress and Challenges of Developing a Vaccine for COVID-19. Front. Immunol..

[46] Xia S., Duan K., Zhang Y., Zhao D., Zhang H., Xie Z., Li X., Peng C., Zhang Y., Zhang W. (2020). Effect of an Inactivated Vaccine Against SARS-CoV-2 on Safety and Immunogenicity Outcomes: Interim Analysis of 2 Randomized Clinical Trials. JAMA.

[47] Rosa M.F.F., da Silva E.N., Pacheco C., Diógenes M.V.P., Millett C., Gadelha C.A.G., Santos L.M.P. (2021). Direct from the COVID-19 crisis: Research and innovation sparks in Brazil. Health Res. Policy Syst..

[48] Dwipayana I.D.A.P. (2020). Efforts in Securing Vaccine for Covid-19 Outbreak in Indonesia. Health Notions.

[49] CGTN China’s Experimental COVID-19 Vaccine Appears Safe, Says Study. https://news.cgtn.com/news/2020-10-08/China-s-experimental-COVID-19-vaccine-appears-safe-says-study-UqbYw2KyDm/index.html.

[50] Saini P. (2020). COVID-19 pandemic: Potential phase III vaccines in development. Appl. Biol. Chem. J..

[51] Sinopharm Says May Be Able to Make Over 1 Billion Coronavirus Vaccine Doses in 2021. Star.

[52] Kim J.H., Marks F., Clemens J.D. (2021). Looking beyond COVID-19 vaccine phase 3 trials. Nat. Med..

[53] Xia S., Zhang Y., Wang Y., Wang H., Yang Y., Gao G.F., Tan W., Wu G., Xu M., Lou Z. (2021). Safety and immunogenicity of an inactivated SARS-CoV-2 vaccine, BBIBP-CorV: A randomised, double-blind, placebo-controlled, phase 1/2 trial. Lancet Infect. Dis..

[54] Calvo Fernández E., Zhu L.Y. (2020). Racing to immunity: Journey to a COVID-19 vaccine and lessons for the future. Br. J. Clin. Pharmacol..

[55] Yoo J.-H. (2021). What We Do Know and Do Not Yet Know about COVID-19 Vaccines as of the Beginning of the Year 2021. J. Korean Med. Sci..

[56] Shivangi S., Meena L.S. (2021). A comprehensive review of COVID-19 in India: A frequent catch of the information. Biotechnol. Appl. Biochem..

[57] Ananthalakshmi V. (2021). The current situation of COVID-19 in India. Brain Behav. Immun. Health.

[58] Bhuyan A. (2021). India begins COVID-19 vaccination amid trial allegations. Lancet.

[59] Moyle P.M., Toth I. (2013). Modern subunit vaccines: Development, components, and research opportunities. ChemMedChem.

[60] Bonavia A., Zelus B.D., Wentworth D.E., Talbot P.J., Holmes K.V. (2003). Identification of a Receptor-Binding Domain of the Spike Glycoprotein of Human Coronavirus HCoV-229E. J. Virol..

[61] Tian J.-H., Patel N., Haupt R., Zhou H., Weston S., Hammond H., Logue J., Portnoff A.D., Norton J., Guebre-Xabier M. (2021). SARS-CoV-2 spike glycoprotein vaccine candidate NVX-CoV2373 immunogenicity in baboons and protection in mice. Nature Commun..

[62] Cheedarla N., Hanna L.E., Buddolla V. (2019). Chapter 7-Functional and Protective Role of Neutralizing Antibodies (NAbs) Against Viral Infections. Recent Developments in Applied Microbiology and Biochemistry.

[63] Krammer F. (2020). SARS-CoV-2 vaccines in development. Nature.

[64] Zhao J., Zhao S., Ou J., Zhang J., Lan W., Guan W., Wu X., Yan Y., Zhao W., Wu J. (2020). COVID-19: Coronavirus Vaccine Development Updates. Front. Immunol..

[65] Rauch S., Jasny E., Schmidt K.E., Petsch B. (2018). New Vaccine Technologies to Combat Outbreak Situations. Front. Immunol..

[66] Vogel F.R., Sarver N. (1995). Nucleic acid vaccines. Clin. Microbiol. Rev..

[67] Restifo N.P., Ying H., Hwang L., Leitner W.W. (2000). The promise of nucleic acid vaccines. Gene Ther..

[68] Jackson N.A., Kester K.E., Casimiro D., Gurunathan S., DeRosa F. (2020). The promise of mRNA vaccines: A biotech and industrial perspective. Vaccines.

[69] Van Nuffel A.M., Benteyn D., Wilgenhof S., Pierret L., Corthals J., Heirman C., Van Der Bruggen P., Coulie P.G., Neyns B., Thielemans K. (2012). Dendritic cells loaded with mRNA encoding full-length tumor antigens prime CD4+ and CD8+ T cells in melanoma patients. Mol. Ther..

[70] Bonehill A., Heirman C., Tuyaerts S., Michiels A., Breckpot K., Brasseur F., Zhang Y., van der Bruggen P., Thielemans K. (2004). Messenger RNA-electroporated dendritic cells presenting MAGE-A3 simultaneously in HLA class I and class II molecules. J. Immunol..

[71] Pardi N., Hogan M.J., Naradikian M.S., Parkhouse K., Cain D.W., Jones L., Moody M.A., Verkerke H.P., Myles A., Willis E. (2018). Nucleoside-modified mRNA vaccines induce potent T follicular helper and germinal center B cell responses. J. Exp. Med..

[72] Pardi N., Hogan M.J., Porter F.W., Weissman D. (2018). mRNA vaccines-a new era in vaccinology. Nat. Rev. Drug Discov..

[73] Zhang C., Maruggi G., Shan H., Li J. (2019). Advances in mRNA vaccines for infectious diseases. Front. Immunol..

[74] Jackson L.A., Anderson E.J., Rouphael N.G., Roberts P.C., Makhene M., Coler R.N., McCullough M.P., Chappell J.D., Denison M.R., Stevens L.J. (2020). An mRNA vaccine against SARS-CoV-2—preliminary report. N. Engl. J. Med..

[75] Moderna (2020). Fact Sheet: Fact Sheet for Recipients and Caregivers.

[76] Baden L.R., El Sahly H.M., Essink B., Kotloff K., Frey S., Novak R., Diemert D., Spector S.A., Rouphael N., Creech C.B. (2021). Efficacy and safety of the mRNA-1273 SARS-CoV-2 vaccine. N. Engl. J. Med..

[77] Ledford H. (2020). Moderna COVID vaccine becomes second to get US authorization. Nature.

[78] Pfizer An Open Letter from Pfizer Chairman and CEO Albert Bourla. https://www.pfizer.com/news/hot-topics/an_open_letter_from_pfizer_chairman_and_ceo_albert_bourla.

[79] Mahase E. (2020). Covid-19: What do we know about the late stage vaccine candidates?. BMJ.

[80] Mahase E. (2020). Covid-19: UK approves Pfizer and BioNTech vaccine with rollout due to start next week. BMJ.

[81] Rollier C.S., Reyes-Sandoval A., Cottingham M.G., Ewer K., Hill A.V. (2011). Viral vectors as vaccine platforms: Deployment in sight. Curr. Opin. Immunol..

[82] Limbach K., Richie T. (2009). Viral vectors in malaria vaccine development. Parasite Immunol..

[83] Tan W.G., Jin H.-T., West E.E., Penaloza-MacMaster P., Wieland A., Zilliox M.J., McElrath M.J., Barouch D.H., Ahmed R. (2013). Comparative analysis of simian immunodeficiency virus gag-specific effector and memory CD8+ T cells induced by different adenovirus vectors. J. Virol..

[84] Sekaly R.-P. (2008). The failed HIV Merck vaccine study: A step back or a launching point for future vaccine development?. J. Exp. Med..

[85] Humphreys I.R., Sebastian S. (2018). Novel viral vectors in infectious diseases. Immunology.

[86] Fausther-Bovendo H., Kobinger G.P. (2014). Pre-existing immunity against Ad vectors: Humoral, cellular, and innate response, what’s important?. Hum. Vaccin. Immunother..

[87] Afkhami S., Yao Y., Xing Z. (2016). Methods and clinical development of adenovirus-vectored vaccines against mucosal pathogens. Mol. Ther. Methods Clin. Dev..

[88] Zhu F.-C., Li Y.-H., Guan X.-H., Hou L.-H., Wang W.-J., Li J.-X., Wu S.-P., Wang B.-S., Wang Z., Wang L. (2020). Safety, tolerability, and immunogenicity of a recombinant adenovirus type-5 vectored COVID-19 vaccine: A dose-escalation, open-label, non-randomised, first-in-human trial. Lancet.

[89] Haque A., Pant A.B. (2020). Efforts at COVID-19 Vaccine Development: Challenges and Successes. Vaccines.

[90] Folegatti P.M., Ewer K.J., Aley P.K., Angus B., Becker S., Belij-Rammerstorfer S., Bellamy D., Bibi S., Bittaye M., Clutterbuck E.A. (2020). Safety and immunogenicity of the ChAdOx1 nCoV-19 vaccine against SARS-CoV-2: A preliminary report of a phase 1/2, single-blind, randomised controlled trial. Lancet.

[91] Roope L.S., Buckell J., Becker F., Candio P., Violato M., Sindelar J.L., Barnett A., Duch R., Clarke P.M. (2020). How should a safe and effective COVID-19 vaccine be allocated? Health economists need to be ready to take the baton. Pharmacoeconomics.

[92] Mahase E. (2020). Covid-19: Vaccine trials need more transparency to enable scrutiny and earn public trust, say experts. BMJ.

[93] Mahase E. (2020). Covid-19: UK approves Oxford vaccine as cases of new variant surge. BMJ.

[94] Sadoff J., Le Gars M., Shukarev G., Heerwegh D., Truyers C., de Groot A.M., Stoop J., Tete S., Van Damme W., Leroux-Roels I. (2020). Safety and immunogenicity of the Ad26. COV2. S COVID-19 vaccine candidate: Interim results of a phase 1/2a, double-blind, randomized, placebo-controlled trial. medRxiv.

[95] Johnson J. Johnson & Johnson Announces Agreement with U.S. Government for 100 Million Doses of Investigational COVID-19 Vaccine. https://www.jnj.com/johnson-johnson-announces-agreement-with-u-s-government-for-100-million-doses-of-investigational-covid-19-vaccine.

[96] Johnson J. Johnson & Johnson Announces European Commission Approval of Agreement to Supply 200 Million Doses of Janssen’s COVID-19 Vaccine Candidate. https://www.jnj.com/johnson-johnson-announces-european-commission-approval-of-agreement-to-supply-200-million-doses-of-janssens-covid-19-vaccine-candidate.

[97] Armin S., Wakil A., Tarbox J., Iwuji K. (2021). COVID-19 vaccination: An attempt to control the pandemic. Southwest Respir. Crit. Care Chron..

[98] Callaway E. (2020). Russia’s fast-track coronavirus vaccine draws outrage over safety. Nature.

[99] Mathew S., Faheem M., Hassain N.A., Benslimane F.M., Thani A.A.A., Zaraket H., Yassine H.M. (2021). Platforms Exploited for SARS-CoV-2 Vaccine Development. Vaccines.

[100] Logunov D.Y., Dolzhikova I.V., Shcheblyakov D.V., Tukhvatulin A.I., Zubkova O.V., Dzharullaeva A.S., Kovyrshina A.V., Lubenets N.L., Grousova D.M., Erokhova A.S. (2021). Safety and efficacy of an rAd26 and rAd5 vector-based heterologous prime-boost COVID-19 vaccine: An interim analysis of a randomised controlled phase 3 trial in Russia. Lancet.

[101] RDIF (2020). Belarus Becomes the FIrst Foreign Country to Register the Sputnik V vaccine. https://rdif.ru/Eng_fullNews/6216/.

[102] Jones I., Roy P. (2021). Sputnik V COVID-19 vaccine candidate appears safe and effective. Lancet.

[103] Chakraborty S., Mallajosyula V., Tato C.M., Tan G.S., Wang T.T. (2021). SARS-CoV-2 vaccines in advanced clinical trials: Where do we stand. Adv. Drug Del. Rev..

[104] Tanne J.H. (2020). Covid-19: FDA panel votes to approve Pfizer BioNTech vaccine. BMJ.

[105] FDA. U.S. Food and Drug Administration Fact Sheet for Healhcare Providers Administering Vaccine (Vaccination Providers). https://www.fda.gov/media/144413/download.

[106] Siegrist C.-A., Lambert P.-H., Bloom B.R., Lambert P.-H. (2016). Chapter 2-How Vaccines Work. The Vaccine Book.

[107] Livingston E.H. (2021). Necessity of 2 Doses of the Pfizer and Moderna COVID-19 Vaccines. JAMA.

[108] FDA. U.S. Food and Drug Administration Pfizer-BioNTech COVID-19 Vaccine Frequently Asked Questions. https://www.fda.gov/emergency-preparedness-and-response/mcm-legal-regulatory-and-policy-framework/pfizer-biontech-covid-19-vaccine-frequently-asked-questions.

[109] Mahase E. (2020). Covid-19: Moderna vaccine is nearly 95% effective, trial involving high risk and elderly people shows. BMJ.

[110] Cavaleri M., Enzmann H., Straus S., Cooke E. (2021). The European Medicines Agency’s EU conditional marketing authorisations for COVID-19 vaccines. Lancet.

[111] Van Tatenhove J.P. (2021). COVID-19 and European maritime futures: Different pathways to deal with the pandemic. Marit. Stud..

[112] Oliver S.E. (2020). The Advisory Committee on Immunization Practices’ Interim Recommendation for Use of Moderna COVID-19 Vaccine—United States, December 2020. Morb. Mortal. Weekly Rep..

[113] Rick J., Thompson A.M., Hsiao J.L., Liao W., Shi V.Y. (2021). Immunosuppressants, Immunomodulators and COVID-19 Vaccines: Anticipating Patient Concerns. J. Dermatolog. Treat..

[114] Widge A.T., Rouphael N.G., Jackson L.A., Anderson E.J., Roberts P.C., Makhene M., Chappell J.D., Denison M.R., Stevens L.J., Pruijssers A.J. (2021). Durability of responses after SARS-CoV-2 mRNA-1273 vaccination. N. Engl. J. Med..

[115] Knoll M.D., Wonodi C. (2021). Oxford–AstraZeneca COVID-19 vaccine efficacy. Lancet.

[116] Wise J. (2021). Covid-19: New data on Oxford AstraZeneca vaccine backs 12 week dosing interval. BMJ.

[117] Yuan P., Ai P., Liu Y., Ai Z., Wang Y., Cao W., Xia X., Zheng J.C. (2020). Safety, Tolerability, and Immunogenicity of COVID-19 Vaccines: A Systematic Review and Meta-Analysis. medRxiv.

[118] Oostvogels L., Kremsner P., Kreidenweiss A., Leroux-Roels I., Leroux-Roels G., Kroidl A., Schunk M., Schindler C., Bosch J., Fendel R. (2020). Phase 1 assessment of the safety and immunogenicity of an mRNA-lipid nanoparticle vaccine candidate against SARS-CoV-2 in human volunteers. medRxiv.

[119] Meo S., Bukhari I., Akram J., Meo A., Klonoff D. (2021). COVID-19 vaccines: Comparison of biological, pharmacological characteristics and adverse effects of Pfizer/BioNTech and Moderna Vaccines. Eur. Rev. Med. Pharmacol. Sci..

[120] CDC (2021). Allergic Reactions Including Anaphylaxis after Receipt of the First Dose of Pfizer-BioNTech COVID-19 Vaccine—United States, 14–23 December 2020. Morb. Mortal. Wkly. Rep..

[121] Shimabukuro T., Nair N. (2021). Allergic Reactions Including Anaphylaxis after Receipt of the First Dose of Pfizer-BioNTech COVID-19 Vaccine. JAMA.

[122] CDC (2021). Allergic Reactions Including Anaphylaxis after Receipt of the First Dose of Moderna COVID-19 Vaccine—United States, 21 December 2020–10 January 2021. Morb. Mortal. Wkly. Rep..

[123] Wadman M. (2020). Public needs to prep for vaccine side effects. Science.

[124] Coffman R.L., Sher A., Seder R.A. (2010). Vaccine adjuvants: Putting innate immunity to work. Immunity.

[125] Garçon N., Segal L., Tavares F., Van Mechelen M. (2011). The safety evaluation of adjuvants during vaccine development: The AS04 experience. Vaccine.

[126] Drexler I., Staib C., Sutter G. (2004). Modified vaccinia virus Ankara as antigen delivery system: How can we best use its potential?. Curr. Opin. Biotechnol..

[127] Delany I., Rappuoli R., De Gregorio E. (2014). Vaccines for the 21st century. EMBO Mol. Med..

[128] Dogra P., Koay E.J., Wang Z., Vahidy F., Ferrari M., Pasqualini R., Arap W., Boom M.L., Sostman H.D., Cristini V. (2021). Do Pandemics Obey the Elliott Wave Principle of Financial Markets?. medRxiv.

[129] Twohey M., Collins K., THomas K. With First Dibs on Vaccines, Rich Countries Have ‘Cleared the Shelves’. https://www.nytimes.com/2020/12/15/us/coronavirus-vaccine-doses-reserved.html.

[130] Rosen B., Waitzberg R., Israeli A. (2021). Israel’s rapid rollout of vaccinations for COVID-19. Isr. J. Health Policy Res..

